# 2,4-Dichloro-1-[1-(2,4-dichloro­benz­yl­oxy)eth­yl]benzene

**DOI:** 10.1107/S1600536809053422

**Published:** 2009-12-16

**Authors:** Jerry P. Jasinski, Ray J. Butcher, C. S. Chidan Kumar, H. S. Yathirajan, B. Narayana

**Affiliations:** a Department of Chemistry, Keene State College, 229 Main Street, Keene, NH 03435-2001, USA; b Department of Chemistry, Howard University, 525 College Street NW, Washington, DC 20059, USA; c Department of Studies in Chemistry, University of Mysore, Manasagangotri, Mysore 570 006, India; d Department of Studies in Chemistry, Mangalore University, Mangalagangotri 574 199, India

## Abstract

In the title compound, C_15_H_12_Cl_4_O, the dihedral angle between the least-squares planes of the two benzene rings is 82.6 (9)°. The dihedral angles between the COC mean plane of the ­oxy group and the two benzene rings are 84.3 (5) and 10.8 (5)°. In the crystal, two weak π–π inter­actions [centroid–centroid distances = 3.9989 (8) and 3.7912 (8) Å] and a C—H⋯π inter­action are observed.

## Related literature

For related structures, see: Yan *et al.* (2007 ▶); Cui *et al.* (2005 ▶); Moratti *et al.* (2007 ▶); Kotila *et al.* (1996 ▶). For compounds related to bis-lactim ethers of cyclic dipeptides, see: Bolte *et al.* (1999 ▶). For catalytic transfer hydrogeno­lysis of benzyl ethers, see: Brigas *et al.* (1999 ▶). For details of theoretical calculations, see: Becke (1988 ▶, 1993 ▶); Frisch *et al.* (2004 ▶); Hehre *et al.* (1986 ▶); Lee *et al.* (1988 ▶); Schmidt & Polik (2007 ▶). For a description of the Cambridge Structural Database, see: Allen (2002 ▶).
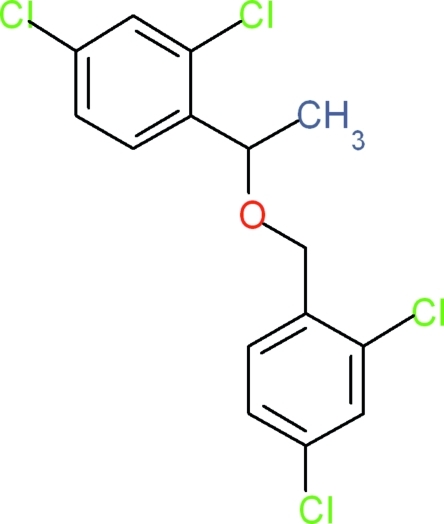


## Experimental

### 

#### Crystal data

C_15_H_12_Cl_4_O
*M* *_r_* = 350.05
Triclinic, 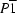

*a* = 9.3755 (4) Å
*b* = 9.9229 (4) Å
*c* = 9.9667 (4) Å
α = 62.313 (3)°
β = 70.246 (4)°
γ = 71.467 (4)°
*V* = 758.22 (5) Å^3^
*Z* = 2
Mo *K*α radiation
μ = 0.77 mm^−1^
*T* = 200 K
0.47 × 0.42 × 0.27 mm

#### Data collection

Oxford Diffraction Gemini diffractometer
Absorption correction: multi-scan (*CrysAlis RED*; Oxford Diffraction, 2007 ▶) *T*_min_ = 0.638, *T*_max_ = 0.812
10547 measured reflections
4961 independent reflections
3334 reflections with *I* > 2σ(*I*)
*R*_int_ = 0.015

#### Refinement

*R*[*F* ^2^ > 2σ(*F* ^2^)] = 0.031
*wR*(*F* ^2^) = 0.086
*S* = 1.02
4961 reflections
182 parameters
H-atom parameters constrained
Δρ_max_ = 0.34 e Å^−3^
Δρ_min_ = −0.25 e Å^−3^

### 

Data collection: *CrysAlis PRO* (Oxford Diffraction, 2007 ▶); cell refinement: *CrysAlis PRO*; data reduction: *CrysAlis PRO*; program(s) used to solve structure: *SHELXS97* (Sheldrick, 2008 ▶); program(s) used to refine structure: *SHELXL97* (Sheldrick, 2008 ▶); molecular graphics: *SHELXTL* (Sheldrick, 2008 ▶); software used to prepare material for publication: *SHELXTL*.

## Supplementary Material

Crystal structure: contains datablocks global, I. DOI: 10.1107/S1600536809053422/is2502sup1.cif

Structure factors: contains datablocks I. DOI: 10.1107/S1600536809053422/is2502Isup2.hkl

Additional supplementary materials:  crystallographic information; 3D view; checkCIF report

## Figures and Tables

**Table 1:** Hydrogen-bond geometry (Å, °)

*D*—H⋯*A*	*D*—H	H⋯*A*	*D*⋯*A*	*D*—H⋯*A*
C12—H12*A*⋯*Cg*1^i^	0.95	2.97	3.8888 (15)	162

Symmetry code: (i) 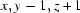. *Cg*1 is the centroid of the C1–C6 ring.

**Fig. 1.**
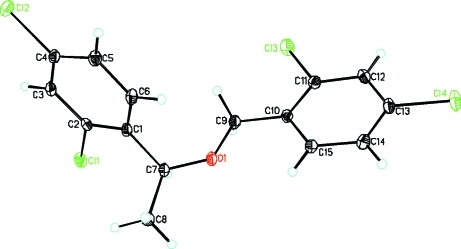
Molecular structure of (I), showing the atom labeling scheme and 50% probability displacement ellipsoids. H atoms are presented as small circles of arbitrary radius.

**Fig. 2.**
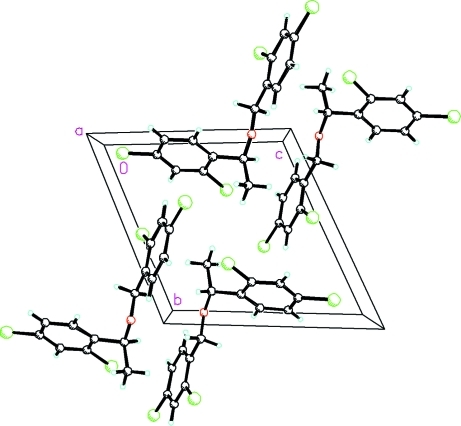
Packing diagram of the title compound, (I), viewed down the a axis.

**Fig. 3.**
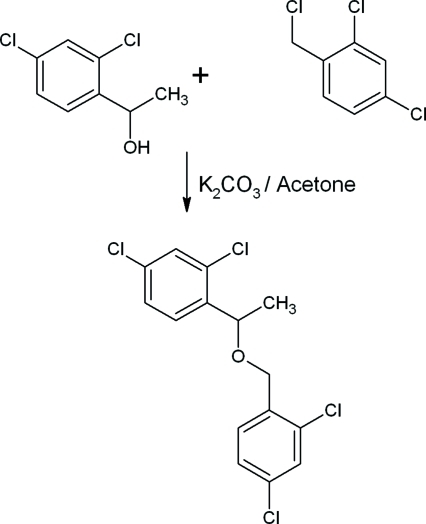
Scheme for the synthesis of 2,4-dichloro-1-{1-[(2,4-dichlorobenzyl) oxy]ethyl}benzene.

## supplementary crystallographic information

### Comment

Ether is a class of chemical compounds which contain an ether group — an oxygen atom connected to two (substituted) alkyl or aryl groups — of general formula R–O–*R*'. Ethers, with their characteristic solvation abilities, excel as inert reaction media in numerous synthetic procedures. However, in practice this usefulness is often tempered by an unfortunate proclivity to facile air oxidation at ambient temperatures which leads to peroxide formation. The structures of the few related compounds *viz*., 4-(benzyloxy)-2-fluorobenzonitrile (Yan *et al.*, 2007), 2-benzyloxy-3-nitropyridine (Cui *et al.*, 2005), 2,6-bis[2-(4-benzyloxyphenyl)ethyl]biphenyl (Moratti *et al.*, 2007), 3-*tert*-butyl-4-methyl-2-phenyl-3-(trimethylsilyloxy)oxetane and 2-(2-benzyloxyphenyl)-3-*tert*-butyl-3-(trimethylsilyloxy)oxetane (Kotila *et al.*, 1996), bis-lactim ethers of cyclic dipeptides: Compounds derived from *cyclo*(Gly-*L*-Val) (Bolte *et al.*, 1999) and 5-benzyloxy-1-phenyltetrazole: catalytic transfer hydrogenolysis of benzyl ethers (Brigas *et al.*, 1999) are already reported. In view of the importance of ethers, the synthesis and crystal structure of the title compound, (I), is reported.

In the title compound, C_15_H_12_Cl_4_O, (I), the dihedral angle between the least squares planes of the two benzene rings is 82.6 (9)° (Fig.1). The angle between the mean planes of the oxy group and the two benzene rings is 84.3 (5)° and 10.8 (5)°, respectively. Each of the two dichloro benzene rings are stacked diagonally along the (011) plane (Fig. 2). While no classic hydrogen bonds are found, weak π–π [*Cg*1···*Cg*1 = 3.9989 (8) Å; 1 - *x*, 2 - *y*, 1 - *z* and *Cg*2···*Cg*2 = 3.7912 (8) Å; 2 - *x*, 1 - *y*, 2 - *z*] and C–H···π [C12–H12A···*Cg*1; Table 1] intermolecular interactions are observed. Bond length and bond angles are within normal ranges (Allen, 2002).

Following geometry optimization using AM1 with MOPAC (Schmidt & Polik, 2007) and density functional theory (DFT) theoretical calculations (Schmidt & Polik, 2007) at the B3LYP/6–31G(*d*) level (Becke, 1988, 1993; Lee *et al.*, 1988; Hehre *et al.*, 1986) with the Gaussian03 program package (Frisch *at al.*, 2004), the dihedral angle between the least squares planes of the two benzene rings becomes 83.6 (3)° (AM1) or 85.9 (6)° (DFT). The angles between the mean planes of the oxy group and the two benzene rings become 86.4 (2) and 3.5 (6)° (AM1) or 88.6 (5) and 5.5 (3)° (DFT), respectively. It is clear that the weak π–π and C—H···π intermolecular interactions do influence crystal packing stability.

### Experimental

A mixture of 1-(2,4-dichlorophenyl)ethanol (0.01 mol, 1.91 g) and 2,4-dichloro-1-(chloromethyl)benzene (0.01 mol, 1.95 g) in 30 ml dry acetone was refluxed over water bath for 6 h (Fig. 3). The crude compound was filtered and recrystallized from ethyl acetate (m.p. 449–451 K). Composition for C_15_H_12_Cl_4_O: C 51.39 (51.46), H 3.42 (3.46).

### Refinement

All of the C-bonded H atoms were placed in their calculated positions and then refined using the riding model with C—H = 0.95 to 1.00 Å, and with *U*_iso_(H) = 1.18–1.49 *U*_eq_(C). The methyl group was allowed to rotate about the C—C bond.

### Crystal data

**Table:**

C_15_H_12_Cl_4_O	*Z* = 2
*M**_r_* = 350.05	*F*(000) = 356
Triclinic, *P*1	*D*_x_ = 1.533 Mg m^−^^3^
Hall symbol: -P 1	Mo *K*α radiation, λ = 0.71073 Å
*a* = 9.3755 (4) Å	Cell parameters from 5527 reflections
*b* = 9.9229 (4) Å	θ = 4.7–32.4°
*c* = 9.9667 (4) Å	µ = 0.77 mm^−^^1^
α = 62.313 (3)°	*T* = 200 K
β = 70.246 (4)°	Chunk, colorless
γ = 71.467 (4)°	0.47 × 0.42 × 0.27 mm
*V* = 758.22 (5) Å^3^	

### Data collection

**Table:**

Oxford Diffraction Gemini diffractometer	4961 independent reflections
Radiation source: fine-focus sealed tube	3334 reflections with *I* > 2σ(*I*)
graphite	*R*_int_ = 0.015
Detector resolution: 10.5081 pixels mm^-1^	θ_max_ = 32.5°, θ_min_ = 4.7°
φ and ω scans	*h* = −13→14
Absorption correction: multi-scan (*CrysAlis RED*; Oxford Diffraction, 2007)	*k* = −14→14
*T*_min_ = 0.638, *T*_max_ = 0.812	*l* = −14→14
10547 measured reflections	

### Refinement

**Table:**

Refinement on *F*^2^	Primary atom site location: structure-invariant direct methods
Least-squares matrix: full	Secondary atom site location: difference Fourier map
*R*[*F*^2^ > 2σ(*F*^2^)] = 0.031	Hydrogen site location: inferred from neighbouring sites
*wR*(*F*^2^) = 0.086	H-atom parameters constrained
*S* = 1.02	*w* = 1/[σ^2^(*F*_o_^2^) + (0.0476*P*)^2^] where *P* = (*F*_o_^2^ + 2*F*_c_^2^)/3
4961 reflections	(Δ/σ)_max_ = 0.001
182 parameters	Δρ_max_ = 0.34 e Å^−^^3^
0 restraints	Δρ_min_ = −0.25 e Å^−^^3^

### Special details

**Table:**

Geometry. All e.s.d.'s (except the e.s.d. in the dihedral angle between two l.s. planes) are estimated using the full covariance matrix. The cell e.s.d.'s are taken into account individually in the estimation of e.s.d.'s in distances, angles and torsion angles; correlations between e.s.d.'s in cell parameters are only used when they are defined by crystal symmetry. An approximate (isotropic) treatment of cell e.s.d.'s is used for estimating e.s.d.'s involving l.s. planes.
Refinement. Refinement of *F*^2^ against ALL reflections. The weighted *R*-factor *wR* and goodness of fit *S* are based on *F*^2^, conventional *R*-factors *R* are based on *F*, with *F* set to zero for negative *F*^2^. The threshold expression of *F*^2^ > σ(*F*^2^) is used only for calculating *R*-factors(gt) *etc*. and is not relevant to the choice of reflections for refinement. *R*-factors based on *F*^2^ are statistically about twice as large as those based on *F*, and *R*- factors based on ALL data will be even larger.

### Fractional atomic coordinates and isotropic or equivalent isotropic displacement parameters (Å^2^)

**Table:**

	*x*	*y*	*z*	*U*_iso_*/*U*_eq_	
Cl1	0.36865 (4)	1.29719 (4)	0.57705 (4)	0.03911 (9)	
Cl2	0.69931 (4)	1.09520 (4)	0.12662 (4)	0.03879 (10)	
Cl3	0.62657 (4)	0.54530 (4)	1.05029 (5)	0.04802 (11)	
Cl4	1.07650 (5)	0.37832 (4)	1.34956 (4)	0.05247 (12)	
O1	0.75079 (10)	1.00830 (9)	0.81884 (9)	0.0308 (2)	
C1	0.65742 (14)	1.12007 (12)	0.58044 (13)	0.0267 (2)	
C2	0.53833 (14)	1.19326 (12)	0.50167 (14)	0.0267 (2)	
C3	0.54921 (14)	1.18650 (12)	0.36200 (13)	0.0281 (3)	
H3A	0.4666	1.2370	0.3100	0.034*	
C4	0.68305 (15)	1.10458 (13)	0.30135 (13)	0.0286 (3)	
C5	0.80410 (15)	1.02893 (14)	0.37548 (14)	0.0323 (3)	
H5A	0.8951	0.9720	0.3324	0.039*	
C6	0.78960 (14)	1.03809 (14)	0.51481 (14)	0.0308 (3)	
H6A	0.8724	0.9869	0.5665	0.037*	
C7	0.64875 (15)	1.13389 (13)	0.72917 (13)	0.0298 (3)	
H7A	0.5404	1.1344	0.7932	0.036*	
C8	0.6967 (2)	1.28197 (15)	0.69213 (17)	0.0428 (3)	
H8A	0.6857	1.2908	0.7892	0.064*	
H8B	0.6307	1.3714	0.6291	0.064*	
H8C	0.8046	1.2794	0.6342	0.064*	
C9	0.69843 (15)	0.86580 (13)	0.89183 (14)	0.0301 (3)	
H9A	0.7024	0.8313	0.8118	0.036*	
H9B	0.5899	0.8804	0.9504	0.036*	
C10	0.79787 (14)	0.74457 (13)	1.00075 (13)	0.0259 (2)	
C11	0.77114 (14)	0.59377 (14)	1.08286 (14)	0.0298 (3)	
C12	0.85496 (15)	0.47887 (14)	1.19003 (14)	0.0330 (3)	
H12A	0.8339	0.3769	1.2447	0.040*	
C13	0.97003 (15)	0.51798 (14)	1.21437 (14)	0.0343 (3)	
C14	1.00324 (16)	0.66490 (15)	1.13334 (15)	0.0354 (3)	
H14A	1.0845	0.6889	1.1500	0.042*	
C15	0.91710 (15)	0.77740 (14)	1.02725 (14)	0.0298 (3)	
H15A	0.9399	0.8787	0.9717	0.036*	

### Atomic displacement parameters (Å^2^)

**Table:**

	*U*^11^	*U*^22^	*U*^33^	*U*^12^	*U*^13^	*U*^23^
Cl1	0.03580 (17)	0.03395 (17)	0.03615 (18)	0.00737 (13)	−0.01065 (14)	−0.01213 (14)
Cl2	0.0446 (2)	0.04732 (19)	0.02785 (16)	−0.01169 (15)	−0.00955 (14)	−0.01509 (14)
Cl3	0.0493 (2)	0.03993 (19)	0.0552 (2)	−0.02003 (16)	−0.02309 (18)	−0.00438 (16)
Cl4	0.0482 (2)	0.0489 (2)	0.0426 (2)	0.00674 (17)	−0.02492 (18)	−0.00436 (16)
O1	0.0381 (5)	0.0265 (4)	0.0249 (4)	−0.0084 (4)	−0.0141 (4)	−0.0018 (3)
C1	0.0305 (6)	0.0236 (5)	0.0224 (5)	−0.0070 (5)	−0.0087 (5)	−0.0033 (5)
C2	0.0273 (6)	0.0194 (5)	0.0264 (6)	−0.0027 (4)	−0.0068 (5)	−0.0042 (4)
C3	0.0283 (6)	0.0260 (6)	0.0254 (6)	−0.0056 (5)	−0.0113 (5)	−0.0028 (5)
C4	0.0360 (7)	0.0277 (6)	0.0201 (5)	−0.0107 (5)	−0.0074 (5)	−0.0042 (5)
C5	0.0292 (6)	0.0326 (6)	0.0281 (6)	−0.0019 (5)	−0.0057 (5)	−0.0097 (5)
C6	0.0259 (6)	0.0350 (6)	0.0258 (6)	−0.0021 (5)	−0.0099 (5)	−0.0072 (5)
C7	0.0351 (7)	0.0269 (6)	0.0232 (6)	−0.0033 (5)	−0.0111 (5)	−0.0054 (5)
C8	0.0641 (10)	0.0304 (6)	0.0389 (7)	−0.0115 (7)	−0.0219 (7)	−0.0092 (6)
C9	0.0342 (6)	0.0281 (6)	0.0258 (6)	−0.0091 (5)	−0.0107 (5)	−0.0044 (5)
C10	0.0271 (6)	0.0265 (5)	0.0206 (5)	−0.0030 (5)	−0.0053 (5)	−0.0080 (5)
C11	0.0296 (6)	0.0302 (6)	0.0287 (6)	−0.0075 (5)	−0.0070 (5)	−0.0097 (5)
C12	0.0342 (7)	0.0268 (6)	0.0297 (6)	−0.0028 (5)	−0.0064 (5)	−0.0073 (5)
C13	0.0338 (7)	0.0342 (6)	0.0262 (6)	0.0051 (5)	−0.0112 (5)	−0.0094 (5)
C14	0.0330 (7)	0.0396 (7)	0.0360 (7)	−0.0053 (6)	−0.0140 (6)	−0.0140 (6)
C15	0.0311 (6)	0.0296 (6)	0.0272 (6)	−0.0052 (5)	−0.0089 (5)	−0.0090 (5)

### Geometric parameters (Å, °)

**Table:**

Cl1—C2	1.7401 (13)	C7—H7A	1.0000
Cl2—C4	1.7398 (12)	C8—H8A	0.9800
Cl3—C11	1.7409 (12)	C8—H8B	0.9800
Cl4—C13	1.7398 (12)	C8—H8C	0.9800
O1—C9	1.4189 (14)	C9—C10	1.5008 (16)
O1—C7	1.4325 (13)	C9—H9A	0.9900
C1—C6	1.3896 (17)	C9—H9B	0.9900
C1—C2	1.3920 (16)	C10—C11	1.3905 (16)
C1—C7	1.5242 (16)	C10—C15	1.3915 (17)
C2—C3	1.3926 (16)	C11—C12	1.3873 (16)
C3—C4	1.3770 (18)	C12—C13	1.3799 (18)
C3—H3A	0.9500	C12—H12A	0.9500
C4—C5	1.3811 (17)	C13—C14	1.3780 (18)
C5—C6	1.3917 (17)	C14—C15	1.3869 (17)
C5—H5A	0.9500	C14—H14A	0.9500
C6—H6A	0.9500	C15—H15A	0.9500
C7—C8	1.5170 (16)		
			
C9—O1—C7	112.73 (9)	C7—C8—H8C	109.5
C6—C1—C2	117.47 (11)	H8A—C8—H8C	109.5
C6—C1—C7	120.34 (10)	H8B—C8—H8C	109.5
C2—C1—C7	122.13 (11)	O1—C9—C10	110.07 (9)
C1—C2—C3	122.11 (11)	O1—C9—H9A	109.6
C1—C2—Cl1	120.19 (9)	C10—C9—H9A	109.6
C3—C2—Cl1	117.69 (9)	O1—C9—H9B	109.6
C4—C3—C2	118.14 (11)	C10—C9—H9B	109.6
C4—C3—H3A	120.9	H9A—C9—H9B	108.2
C2—C3—H3A	120.9	C11—C10—C15	117.12 (11)
C3—C4—C5	122.02 (11)	C11—C10—C9	120.55 (10)
C3—C4—Cl2	118.89 (9)	C15—C10—C9	122.31 (10)
C5—C4—Cl2	119.09 (10)	C12—C11—C10	123.01 (11)
C4—C5—C6	118.40 (12)	C12—C11—Cl3	118.03 (9)
C4—C5—H5A	120.8	C10—C11—Cl3	118.96 (9)
C6—C5—H5A	120.8	C13—C12—C11	117.65 (11)
C1—C6—C5	121.85 (11)	C13—C12—H12A	121.2
C1—C6—H6A	119.1	C11—C12—H12A	121.2
C5—C6—H6A	119.1	C14—C13—C12	121.51 (11)
O1—C7—C8	106.63 (10)	C14—C13—Cl4	119.22 (10)
O1—C7—C1	111.59 (10)	C12—C13—Cl4	119.27 (10)
C8—C7—C1	110.86 (10)	C13—C14—C15	119.47 (12)
O1—C7—H7A	109.2	C13—C14—H14A	120.3
C8—C7—H7A	109.2	C15—C14—H14A	120.3
C1—C7—H7A	109.2	C14—C15—C10	121.21 (11)
C7—C8—H8A	109.5	C14—C15—H15A	119.4
C7—C8—H8B	109.5	C10—C15—H15A	119.4
H8A—C8—H8B	109.5		
			
C6—C1—C2—C3	−0.25 (17)	C2—C1—C7—C8	−83.23 (14)
C7—C1—C2—C3	176.98 (10)	C7—O1—C9—C10	−172.27 (9)
C6—C1—C2—Cl1	179.61 (9)	O1—C9—C10—C11	−178.34 (11)
C7—C1—C2—Cl1	−3.16 (15)	O1—C9—C10—C15	3.20 (17)
C1—C2—C3—C4	−0.16 (17)	C15—C10—C11—C12	1.43 (19)
Cl1—C2—C3—C4	179.98 (8)	C9—C10—C11—C12	−177.11 (12)
C2—C3—C4—C5	0.65 (17)	C15—C10—C11—Cl3	−178.37 (10)
C2—C3—C4—Cl2	−179.79 (8)	C9—C10—C11—Cl3	3.10 (16)
C3—C4—C5—C6	−0.72 (18)	C10—C11—C12—C13	−0.2 (2)
Cl2—C4—C5—C6	179.73 (9)	Cl3—C11—C12—C13	179.57 (10)
C2—C1—C6—C5	0.18 (18)	C11—C12—C13—C14	−1.24 (19)
C7—C1—C6—C5	−177.10 (11)	C11—C12—C13—Cl4	179.17 (10)
C4—C5—C6—C1	0.28 (19)	C12—C13—C14—C15	1.4 (2)
C9—O1—C7—C8	166.43 (10)	Cl4—C13—C14—C15	−178.97 (10)
C9—O1—C7—C1	−72.37 (12)	C13—C14—C15—C10	−0.2 (2)
C6—C1—C7—O1	−24.78 (15)	C11—C10—C15—C14	−1.21 (18)
C2—C1—C7—O1	158.07 (10)	C9—C10—C15—C14	177.29 (12)
C6—C1—C7—C8	93.92 (14)		

### Hydrogen-bond geometry (Å, °)

**Table:**

Cg1 is the centroid of the C1–C6 ring.

**Table:**

*D*—H···*A*	*D*—H	H···*A*	*D*···*A*	*D*—H···*A*
C12—H12A···Cg1^i^	0.95	2.97	3.8888 (15)	162

Symmetry codes: (i) *x*, *y*−1, *z*+1.
